# Application of Computed Tomography Processed by Picture Archiving and Communication Systems in the Diagnosis of Acute Achilles Tendon Rupture

**DOI:** 10.1155/2016/6043638

**Published:** 2016-12-18

**Authors:** Hai-Peng Xue, Xin-Wei Liu, Jing Tian, Bing Xie, Chao Yang, Hao Zhang, Da-Peng Zhou

**Affiliations:** Department of Orthopedics, General Hospital of Shenyang Military Area Command of Chinese PLA, Rescue Center of Severe Wound and Trauma of Chinese PLA, Shenyang, Liaoning 110016, China

## Abstract

The applications of CT examination in the diagnosis of the acute Achilles tendon rupture (AATR) were investigated. A total of 36 patients with suspected acute Achilles tendon rupture were tested using physical examination, ultrasound, and 3DCT scanning, respectively. Then, surgery was performed for the patients who showed positive result in at least two of the three tests for AATR. 3DVR, MPR, and the other CT scan image processing and diagnosis were conducted in PACS (picture archiving and communication system). PACS was also used to measure the length of distal broken ends of the Achilles tendon (AT) to tendon calcaneal insertion. Our study indicated that CT has the highest accuracy in diagnosis of acute Achilles tendon complete rupture. The length measurement is matched between PACS and those actually measured in operation. CT not only demonstrates more details directly in three dimensions especially with the rupture involved calcaneal insertion flap but also locates the rupture region for percutaneous suture by measuring the length of distal stump in PACS without the effect of the position of ankle. The accuracy of CT diagnosis for Achilles tendon partial rupture is yet to be studied.

## 1. Introduction

Acute Achilles tendon rupture is the most common tendon injures of lower limbs [1]. Physical examination, color Doppler ultrasound (US), and magnetic resonance imaging (MRI) have high rates of diagnosis, but each one has its drawbacks and limitations, so there is still no golden standard for diagnosis. Thompson's sign test is not very obvious in some patients with AATR for the intact plantar tendon or more proximal rupture. Ultrasound is often chosen to diagnose AATR, because of high accuracy and being economical and easy to use. However, complete rupture is usually misdiagnosed by ultrasound as partial rupture which will influence the strategy of treatment [2]. MRI also has high accuracy, but it may have few days' appointment for examination, and some authors even suggest MRI is unnecessary for diagnosing AATR [3].

CT is usually used for checking lungs, brain, and internal organs. Three-dimensional computed tomography (3DCT) has been used to diagnose the osteopathy for its high spatial resolution which can appraise bones in any views. It has been reported that 3DCT was used for tendon inspection two decades ago [4], but the results appeared unsatisfactory due to technology limitations. Thus, CT diagnosis of soft tissue has not been widely used. However, CT technology has been developing rapidly in recent years. Hardware has developed from the 4 rows scan to 256 MDCT and even dual energy CT [5] as well as gemstones CT [6]. Postprocessing technology has also been improved greatly such as the emergence of shaded surface display (SSD), maximum intensity projection (MIP), multiplanar reconstruction (MPR), three-dimensional-volume rendering (3DVR) [7–11], and kinematic 4DCT [12]. The new technologies not only reduce the diagnostic time significantly, but also demonstrate more distinction and intuitive information than before. Recently, some authors have reported the diagnosis of tendon disease with computed tomography [13, 14]. However, the accuracy of CT technology for diagnosis of acute Achilles tendon rupture has not been reported.

PACS has been widely used in most of large-scale hospitals. It can receive and store archive images from medical imaging modalities (including CT, MR, CR, DX, and any other DICOM device, except US) and distributes them to DICOM devices [15]. DICOM data saved in PACS servers will not deform images. The images can be stored for a long time and can be retrieved anytime in any computer where PACS client has been installed. Surgeons trained in a few hours can process DICOM by PACS like radiologists and are able to get more useful information and details of injures directly. In this work, we expect to explore the advantages and disadvantages of CT diagnosis processed by PACS of AATR.

## 2. Materials and Methods

### 2.1. Patients Selection and Diagnosis

From February 1, 2014, to May 1, 2016, patients who had a history of acute sudden pain of Achilles tendon were included. Cases such as opening injure, Achilles tendinitis, and chronic rupture were excluded. The patients were examined by US and 3DCT scanning after one orthopedist finished the physical examination. And then, another orthopedist processed the DICOM data with 3DVR and MPR modules of PACS and got a CT diagnosis.

### 2.2. CT Examination

GE LightSpeed VCT, KVP 120 kV, X-ray tube current 200–230 mA, slice thickness 0.625 mm, spacing between slices 5.0, and 64 Spiral CT were used in this study. Dual energy CT can better display calcium deposits in the blood vessels, but not as good as ordinary CT on showing the tendon [16]. Gemstone CT can effectively reduce the shadow of implants, but not improve tendon images [17]. As we all know, CT scanning parameters are different for different tissues. The greater the tube current the better the image which displays the details of the tissues while the amount of radiation is larger. There are no common CT conditions for the tendon scanning. The parameters for the bone scanning are used for tendon scanning though a clear image of Achilles tendon can be obtained [13, 14]. Hopefully, scholars can design the parameters specifically for Achilles tendon scanning. So the radiation absorption can be reduced and clear images can be obtained.

### 2.3. PACS

**Figure 1:**
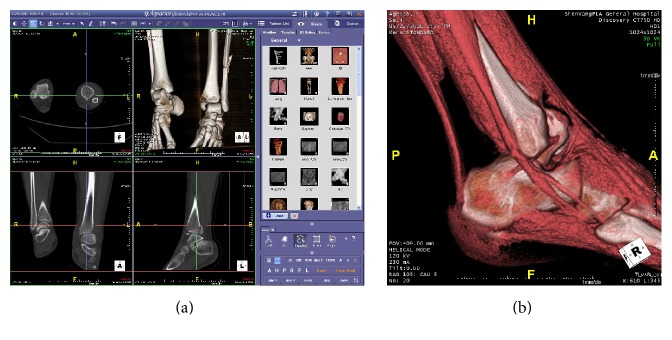
(a) PACS with four windows in left display three orthogonal MRP images and one 3DVR image, and module list in right prepare for displaying various tissues. (b) Select the module we have created before to display bone and tendon in one window. The fibula, calcaneus, peroneus longus and brevis muscles, and Achilles tendon can be seen clearly.

The information of patient scanned by CT was saved as DICOM and was translated to PACS (Aquarius Net, Terarecon, Foster City, CA, USA) servers. We can retrieve those data by searching ID or names in PACS which has many useful modules like 3DVR, MPR, MIP, VR, CPR, and other image processing functions. Each module has many templates. We can switch and demonstrate various tissues in one window by choosing different templates of 3DVR. We can also create a new template to demonstrate many tissues in one picture (Figure 1(a)). In this research, we have created template which can display bone and tendon at the same time to observe the relationship between them. The measurement function allows us to measure the distance between any points or lines in 2D or 3D images and can be accurate to 0.1 mm.

### 2.4. 3DVR Postprocessing

3DVR Reconstruction is the most common CT three-dimensional displaying method [18]. The attenuation values (or Hounsfield units) are different for different tissue at CT scan. HU of air is defined as −1000, water HU is defined as 0, the fat is about −100 HU, muscle 40 HU, Achilles 100 HU, and bone 200–500 HU [13]. Different tissues can be displayed in one image distinguishingly when different HU threshold values are assigned to different colors. The front of the Achilles tendon is fat and the skin is at the back. A three-dimensional image of the tendon and bone can be obtained after hiding the skin and fat based on the HU values. After we got the data of patients by PACS, choose the bone 3DVR template (window width 500, window level 400, opacity 1.0, and white color) and add a tendon tissue bar (window width 200, window level 100, opacity 0.8–1.0, and red color), or choose the template we had created before. It is important to note that the voltage can affect HU value. The higher the voltage is, the higher the HU value is [19], so fine-tune is needed to get clear outline of Achilles tendon (Figure 1(b)).

### 2.5. MPR Postprocessing

**Figure 2:**
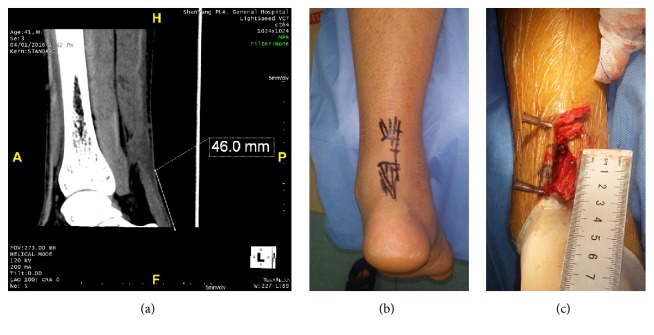
41-year-old male. (a) Adjusting the axis of images though the MPR module and measuring the length of distal part. (b) Locating the rupture position by the distance from the calcaneal insertion of AT measured in CT MPR images. (c) Measuring the length of distal part intraoperation directly.

When a patient lays on his back for CT scan, legs are in a swing state. The coronal and sagittal images based on torso axis are not lower limb axial images (Figure 1(a)). The coronal and sagittal images of lower limb can be obtained through MPR reconstruction by adjusting the angle of the axis. The images of coronal, sagittal, and tangential are shown in mutually perpendicular plane, respectively. The axis of each plane is adjusted to get real axial, sagittal, and coronal images of AT. Better contrast between tendons and other soft tissues can be obtained by window width and window level through MPR module. You can also adjust the axis to vertical or horizontal to the tibia, tendons, or other tissues to reduce the position requirements for CT scan. More details of the damage can also be learned from the different levels [9]. We can observe the continuity and tension of AT in 3DVR and MPR images and get a CT diagnosis. If the diagnosis is rupture, the length of distal broken ends to tendon calcaneal insertion would be measured (Figure 2(a)).

### 2.6. Treatments

A surgery will be conducted if a patient shows positive result in at least two of those three tests: (A) Thompson's sign test in clinical examination; (B) complete rupture diagnosed by ultrasound; (C) complete rupture diagnosed by CT. The length of distal stump measured in CT MPR images was used as a reference in a mini-incision (Figure 2(b)). An operation would be conducted to fix the rupture tendons, and the real length of distal stump would be measured in the operation (Figure 2(c)). An above-knee cast with ankle plantar flexion is placed after the operation for 3 weeks and then shifted to below-knee cast for another 3 weeks.

## 3. Result 

### 3.1. Patients Information

There are 33 males and 3 females in the study. The oldest is 81 years old, and the youngest is 17 years old. The average age is 37.6 years. The shortest time between diagnosis and injury was 0.5 hours and the longest time was 48 hours. For the causes of injuries, 11 patients were injured from playing basketball, 6 patients were injured during playing badminton, 4 patients were injured due to playing jump rope, 4 patients were injured because of playing soccer, 3 patients were injured during running, 4 patients were injured in the training, and 5 patients were injured due to other causes.

### 3.2. Accuracy of CT Diagnosis

As listed in Table 1, there are total of 36 patients. 33 patients showed positive result in Thompson's sign test. 4 were diagnosed as Achilles tendon partial rupture (PR) and 32 were diagnosed as complete Achilles tendon rupture (CR) in Doppler ultrasound diagnosis. CT results showed all 36 patients had acute Achilles tendon complete rupture. In the operation, all the Achilles tendons were completely ruptured. Based on these results, CT diagnosis is the most accurate method to diagnose acute Achilles tendon complete rupture. Diagnostic accuracy of CT for acute Achilles tendon partial rupture is to be studied.

### 3.3. Accuracy of CT Measurement

**Figure 3:**
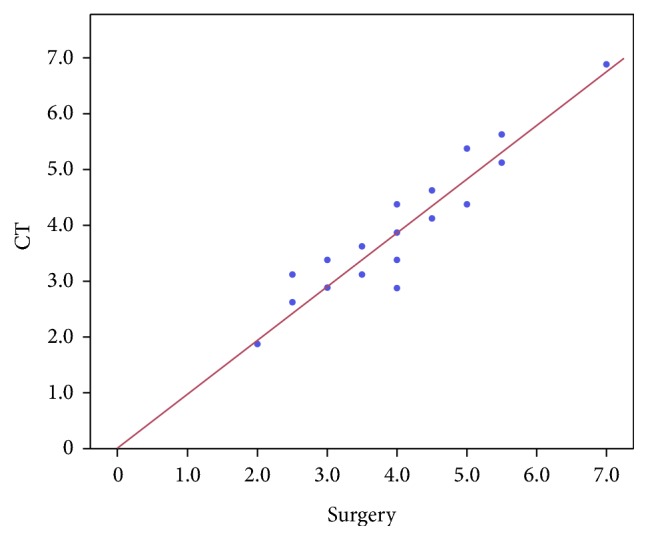
The correlation between CT measurements and the measurements in surgeries on the length of distal stump.

The length of distal stump (LODS) measured in CT is 3.71 ± 1.16 cm (range 0–7.0 cm), and LODS measured intraoperation is 3.83 ± 1.17 (range 0–7.2 cm) (Figure 3). Pearson correlation coefficients show a high correlation (*r* = 0.963, *p* < 0.01) (Table 2).

## 4. Discussion 

AATR is the most common tendon of lower limbs [1], although the AT is one of the strongest tendons of human [20]. It usually happens in patients between 30 and 40 years old [21] and significantly more men than women [22]. The incidence rate has trended higher [23, 24] recently. Rupture is located in the middle distance at the tendon insertion 2–6 cm [25]. In our study, the ratio of men to women is close to 11 : 1 with an average of 37.6 years old. Most incidences happened when patients took part in various sports activities who are middle-aged and fat. The distance between the rupture site to the tendon stump position has an average of 3.8 cm as some other studies reported.

Clinical examination is the first and most important step to diagnose the Achilles ruptures: ecchymosis and swelling occur rapidly in the first 24 hours after injury. A palpable gap can be touched at the rupture site and plantar flexion power is more often diminished [26]. Thompson's sign test has been a routine examination since it is described by Thompson and Doherty in 1962 [27]. However, Thompson's sign test is not very obvious for the intact plantar tendon, and the diminished plantar flexion strength may be less with a more proximal rupture [28], and the palpable gap will be overlooked with soft tissue swelling. In this study, Thompson's sign was weakly positive for 3 patients, and these 3 patients had the plantar tendons intact. Therefore, some other diagnostics are necessary as supplements of Thompson's sign test. Doppler ultrasound and magnetic resonance imaging are recognized to be the most useful technique to diagnose the AATR [29, 30].

Margetić et al. [2] study showed that ultrasound has an excellent result. But 8 patients with complete rupture had been diagnosed as partial rupture, though it was found that only 2 patients had partial rupture in the operation. The misdiagnosis might result in entirely different therapeutic strategies and prognosis. Coincidentally, the accuracy of ultrasound diagnosis will be also influenced by experience and some other subjective factors of the sonographer. Otherwise, orthopedist can only be given a result of the diagnosis such as Achilles tendon complete rupture. There are not any other details like the region, range of rupture, or the relationship with the bone around it which could be observed by imaging examination.

**Figure 4:**
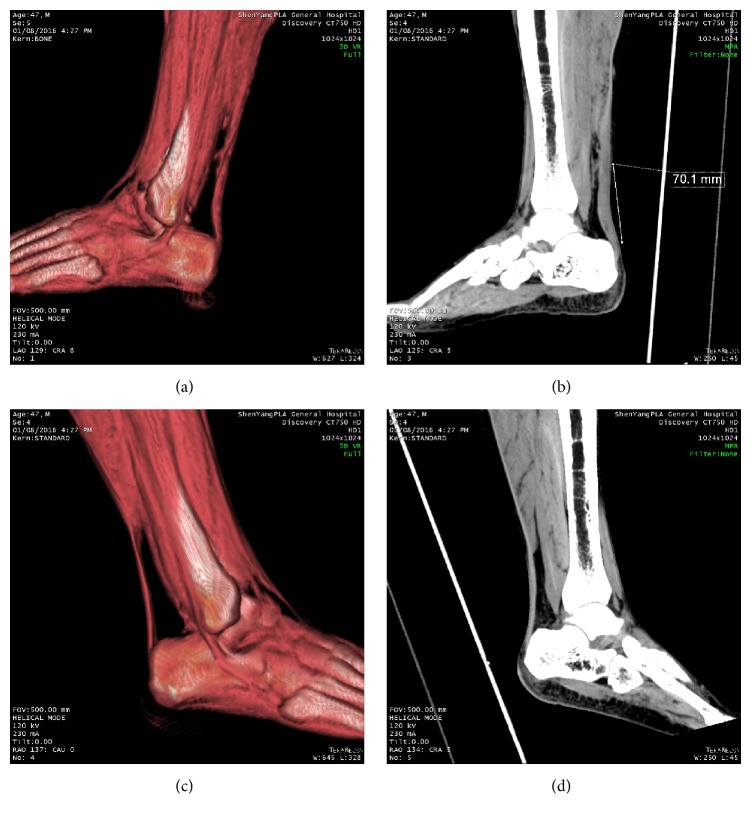
Male patient, 47 years old, was injured while playing badminton. (a) The rupture of Achilles tendon is close to near end and the distance to Achilles point is 7 cm. 3DVR outline image shows the broken ends of the tendon have thickened. (b) Ipsilateral MPR images showed distal tendon thickened and slightly bent due to tension disappearance. Contralateral 3DVR image (c) and MPR image (d) showed good tendon tension but thinner compared with the patients. It is easy to find the difference with affected side.

In this study, 4 patients were diagnosed by ultrasound as partial rupture. but Thompson's test showed positive sign, and they were diagnosed by CT as Achilles tendon complete rupture. It can be seen in CT 3DVR images that the tendon was thickening, rather than thinning or missing at broken ends (Figure 4(a)). They were caused by the overlap of broken end and hematomas near the surrounding areas, where the HU value is approximate to tendons. So it looks like Achilles Tendinitis or old Achilles tendon injury [13]. Furthermore, 3DVR images showed bending and thickening of the tendon, and MPR images were even more obvious because the tension disappears and the distal part of tendon retreats slightly for the AATR (Figure 4(b)).

In addition, the accuracy can be even improved if comparing with the contralateral limb (Figures 4(c) and 4(d)). This can be done by increasing the scanning window and getting data of both lower extremities. This does not increase radiation exposure time or radiation damage because it is only one scan and the absorbed radiation for distal limb is much less than the abdomen. The only cost is to need more storage to save the data.

MRI has a higher accuracy than ultrasound in the diagnosis of Achilles tendon rupture, especially chronic ones [30, 31]. But Garras et al. [3] suggested MRI is unnecessary for diagnosing AATR because it would take so many days to obtain MRI scanning after the injury, which is time consuming and expensive and may lead to treatment delays. MRI can even be contraindicated if patients have metal hardware or a history of claustrophobia or obesity [7]. In our hospital, MRI check has been occasionally used as diagnosis for AATR. Patients usually need to wait for 1–3 days. After that soft tissue is at the peak of edema and the distance of broken end increases due to broken end shrinking back gradually. These caused difficulty of microsurgery, and also increased risk of incision healing. Therefore, we do not use MRI diagnosis unless other examinations do not work. By contrast, CT is more efficient. It is easily to take CT scanning, and we can get the information by PACS quickly, when the patients may not leave the scanning room.

**Figure 5:**
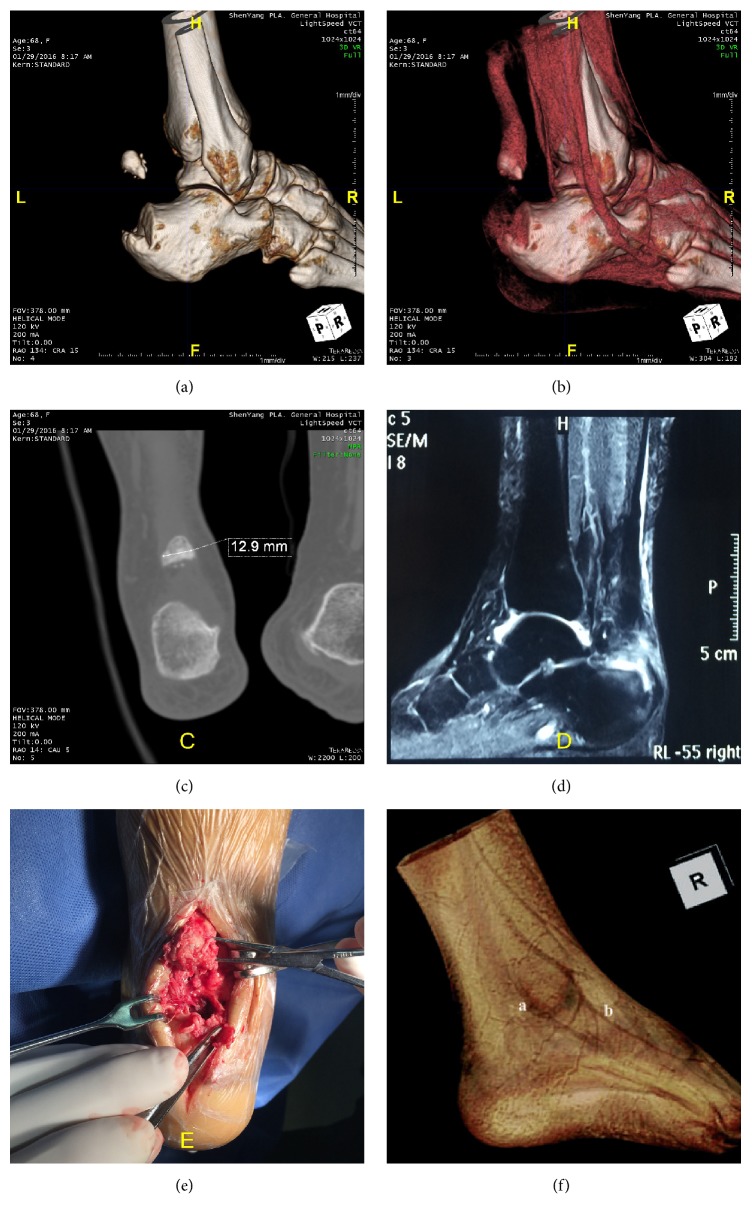
Female patient, 68 years old, hurt in the shower. (a) 3DVR image displays free bone at the back of calcaneus. (b) Widened tendon tissue image, visible Achilles tendon insertion avulsion fragment, Achilles tendon tension disappearing, and visible small tear of the Achilles tendon stump. (c) Design procedures based on bone size through PACS System. (d) The avulsion fragment cannot be seen in MRI images. (e) Achilles tendon avulsion of bone and torn tendon of distal stump observed in the surgery. (f) MRI 3DVR processed image, hard to get 3D image of Achilles tendon due to similar water content in Achilles tendon and the surrounding tissue (image from [32]).

Clinicians can use PACS System observing any position of an injury intuitively in 3DVR images, rather than getting only one conclusion from ultrasound and hard to understand details of the damage. CT's advantage is more significant when Achilles tendon calcaneal insertion breaks and combined with avulsed bone (Figures 5(a)–5(e)). MRI data can also be processed using 3DVR, but it is hard to get clearer images. It is difficult to hide the surrounding tissues from tendon based on HU values, because water content in the Achilles tendon is similar to water content in the surrounding tissues and they have similar HU values [32] (Figure 5(f)).

Minimally invasive percutaneous suture technique has been accepted by the majority of doctors because it can greatly reduce the complications of the surgical incision of Achilles tendon problems [33–37]. The precise positioning of the broken end is the basis of minimally invasive surgery. After Achilles tendon ruptures, it is difficult to determine the accurate position because proximal muscles contract. Despite the fact that distal tendinous length has smaller change, it is still difficult to accurately locate the broken position because the tendon sheath remained relatively intact and distal tendon can slide up and down in the lumen of the sheath as well as due to local swelling [38]. Ultrasound can position broken end in surface of projection location, but errors may also occur with ankle joint plantarflexion or dorsiflexion [39]. Some doctors used ultrasound for positioning during surgeries, but they need to have ultrasound knowledge which makes it hard to promote this practice [40]. Accordingly, it was mostly relying on the doctor's experience to determine the location of broken end.

Another significant advantage of CT in the diagnosis of AATR is to determine the rupture location. The length of distal stump of Achilles tendon is relatively stable. We can locate the rupture position with the distance to the calcaneal insertion measured predetermined in CT MPR images whenever ankle joint is plantarflexion or dorsiflexion. So the accurate measurement is very helpful to locate the rupture position in the surgeries. Figure 3 indicated that there is high correlation between CT measurements and the measurements in the surgeries on the distal stump length.

In this study, there were two orthopedists to make the clinical diagnosis and CT diagnosis independently to eliminate subjective factors, but CT still showed excellent accuracy. Our sample size is small, and there is no acute Achilles tendon partial rupture. So the result may be more convinced when the sample size is larger and contains more types of rupture.

Some scholars also believe that there is no difference between normal image of Achilles tendon and the image of the partial rupture of Achilles tendon [13]. We did not see Achilles tendon partial rupture in our study. Thus, CT diagnosis of acute Achilles tendon partial rupture is yet to be studied.

In some cases, CT images of AATR are similar with Achilles Tendinitis or old Achilles tendon injury, so medical history is necessary for diagnosis.

## 5. Conclusions

CT diagnosis has higher accuracy than physical examination or color US for the acute Achilles tendon complete rupture. The details of the injury can be reviewed readily based on CT postprocessing data using PACS System. The relationship of Achilles tendon and the surrounding bone tissues can be shown when the broken end involves the avulsed fragment. The position and length can be accurately measured by PACS, which can provide strong support for minimally invasive surgery. But the accuracy of CT diagnosis for Achilles tendon partial rupture is yet to be studied.

## Figures and Tables

**Table 1:** Comparison of Thompson's sign test, US, CT diagnosis, and intraoperation findings.

		Thompson's sign test		Total
		Positive	Negative or WP	
		33	3	36
US	CR	29	3	32
	PR	4	0	4
CT	CR	33	3	36
	PR	0	0	0
IO	CR	33	3	36
	PR	0	0	0

WP: weakly positive; CR: complete rupture; PR: partial rupture; IO: intraoperation.

**Table 2:** The Pearson correlation coefficient of the LODS measured by CT and surgeries.

Correlations			
		CT	IO
CT	Pearson correlation	1	0.963
	Sig. (2-tailed)		0.000
	*N*	36	36

IO	Pearson correlation	0.963	1
	Sig. (2-tailed)	0.000	
	*N*	36	36
